# Association rule mining of aircraft event causes based on the Apriori algorithm

**DOI:** 10.1038/s41598-024-64360-6

**Published:** 2024-06-11

**Authors:** Huaqun Chen, Minghui Yang, Xie Tang

**Affiliations:** 1 Air Traffic Management Department, Civil Aviation Flight University of China, Guanghan, Sichuan China; 2 Sichuan Highway Planning Survey Design Institute Co., Ltd., Sichuan, Chengdu China

**Keywords:** Data mining, Aircraft event, Causes of unsafe events, Association rule, Modified Apriori algorithm, Aerospace engineering, Computer science

## Abstract

To reveal complex causes of aircraft events, this paper aims to mine association rules between the trigger probability and relative strength via a modified Apriori algorithm. Clustering is adopted for data preprocessing and TF–IDF value calculation. Causative item sets of aircraft events are obtained based on the accident causation 2–4 model and are coded to establish code indicators. By avoiding the use of statistical methodologies to resolve not-a-number (NaN) values for altering the interrelations among causes, an enhancement in the Apriori algorithm is proposed by considering frequent items. By extracting frequent patterns, in this paper, all the association rules that satisfy three perspectives (support, confidence and lift) are determined by constantly generating and pruning candidate item sets. A network graph is used to visualize the association rules between different unsafe events and all types of causes. Finally, 9835 representative pieces of data, including general unsafe events, general incidents and serious incidents from the Southwest Air Traffic Management Bureau, are selected for analysis. The results show that improper energy allocation, poor conflict resolution ability, inadequate onsite management duties, adoption of a luck mentality, and occurrence of controller oversight are highly correlated with general unsafe events, and failure to rectify incorrect recitation is notably correlated with general incidents, while inadequate manual promotion, lack of conflict judgement and insufficient safety management are strongly correlated with serious incidents. This study quantitatively reveals the potential patterns and characteristics of mutual interactions among various types of historical aircraft events and highlights directions for controllable prevention and prediction of aircraft events.

## Introduction

**Figure 1:**
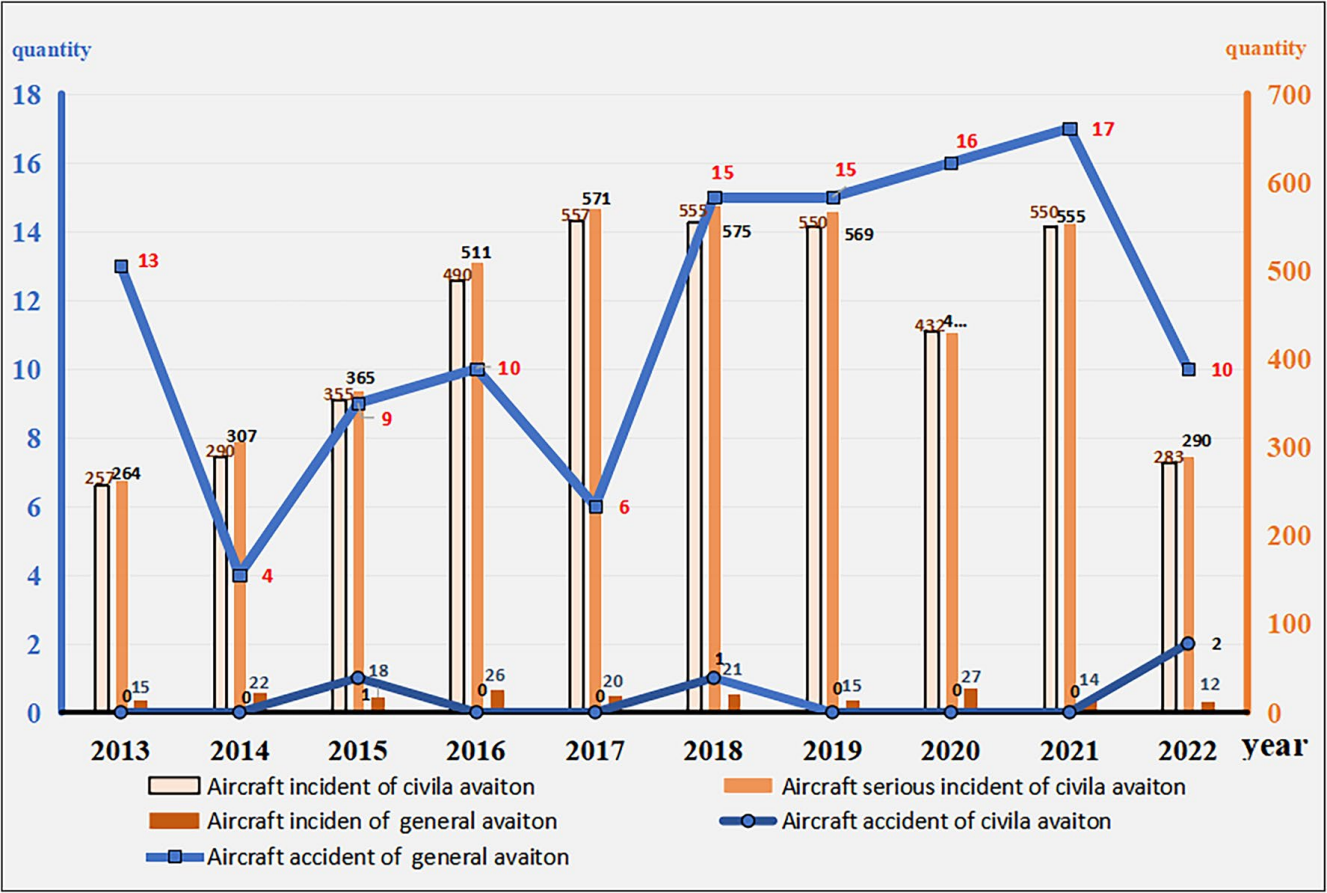
Civil and general aviation aircraft accidents and incidents in China from 2013 to 2022.

Association analysis of aircraft accident causation involves deriving probabilities of event types based on historical event causes after learning and training by considering past incidents. This method serves as a crucial means for preventing and predicting unsafe events or accidents^1–3^. With the rapid development of civil aviation in China, the number of flights has significantly increased, and air transport has become a major link in international communication and domestic economic development. However, airline networks are complex, organizational structures are vast, regional differences are notable, and unsafe events frequently occur. According to statistics of the Civil Aviation Administration of China Safety, the accident occurrence ratio reached 0.29%, the incident occurrence ratio reached 11.39%, and general unsafe events accounted for 88.32% of the total aircraft events from 2013 to 2022, as shown in Fig. 1.

**Figure 2:**
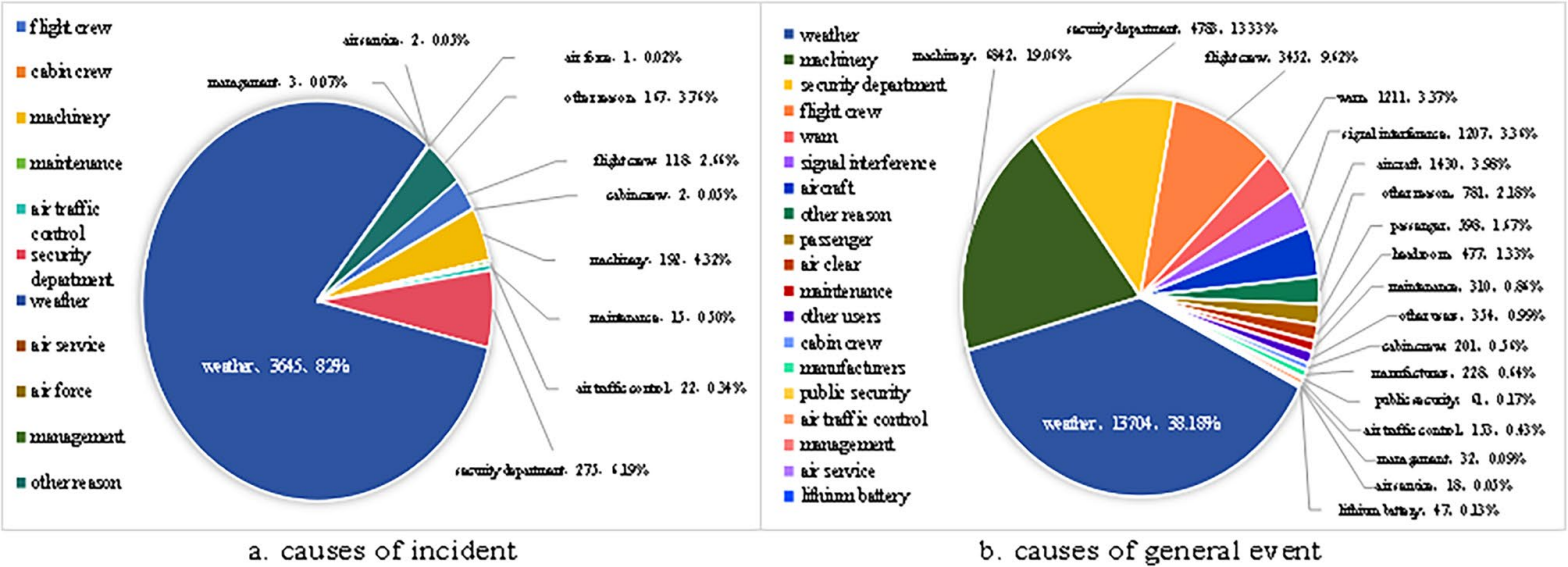
Causes of incidents and general unsafe events in China from 2019 to 2022.

Figure 1 indicates that the occurrence of accidents and incidents linearly increased from 2013 to 2019. Due to the COVID-19 pandemic, the number of flight accidents and incidents plummeted from 2020 to 2022. However, the number of general unsafe events has increased to approximately 10,000 per year. Aircraft event investigations revealed that the number of causes underlying unsafe events and incidents exceeded 20, and the proportions are shown in Fig. 2.

According to the results of these investigations, many unsafe events are typically caused by multiple factors, including human factors, equipment usage, management systems, and internal/external environments. However, the characteristic indicators leading to aircraft incidents exhibit discreteness, constrained by dynamically extracted factors. This constraint prevents precise quantification, leaving the determined causes restricted to a qualitative level, thereby affecting the reliability of the analysis and prediction results. Hence, it is necessary to reveal meaningful connections hidden within investigation data of aircraft incidents by employing machine learning to establish association rules. Association analysis aims to assess the correlations among various incident factors and event types comprehensively and systematically. Subsequently, preventive measures to reduce the occurrence of similar events or accidents can be adopted.

This paper aimed to introduce a data-mining technique. A substantial amount of incident investigation data was analysed in depth. Comprehensive and accurate correlational information was derived, and inherent correlations leading to event causes were revealed. By establishing various association rules, greater insights were obtained. A pattern of potential correlations leading to unsafe aircraft incidents was revealed. Finally, the advantages of real-time high-speed data streaming were exploited, and unsupervised and supervised learning approaches were adopted. The latest information and insights were provided so that investigators can perceive and detect clues and anomalies related to incidents in a timely manner.

### Literature review

Accident causation theory is an essential method for studying the occurrence and development of accidents^4^. Experts worldwide have studied factors influencing safety events across industries, such as coal mining^5^, road traffic^6^, and railway transportation^7^, by developing various event causation models and distinct theories of accident causation. As of December 2023, there are more than fifty distinct accident causation models, which can be categorized into linear, contagion and systemic accident causation models^8^. Systemic accident causation models are the most widely adopted and consider the entire accident as a whole rather than analysing individual causal factors^9^.

Lenné et al.^10^ analysed 169 general aviation accidents in Australia using the HFACS model and revealed a positive correlation between crew violations and deficiencies in crew resource management. Li et al.^11^ statistically analysed the causes of 41 civil aviation accidents in Taiwan from 1999 to 2006 using the HFACS model, and a relationship diagram of the considered causes was derived by using chi-square tests. Chang et al.^12^ examined human factor risk elements in runway incursion-related accidents utilizing an improved SHEL-SHELLO model. Kharoufah et al.^13^ conducted a random study of more than 200 commercial aviation transport accidents between 2000 and 2016 and used chi-square tests to detect the factors influencing these accidents. Stojiljković et al.^14^ utilized systematic human error reduction and prediction methods to identify 55 errors that occurred among 30 pilots over 10 years, and a hierarchical task analysis and classification method was established based on pilot tasks to analyse the probability and consequences of error occurrence. Chen et al.^15^ analysed human factors in 484 aviation accidents from 1999 to 2012 and identified causal relationships among human and other factors. Sun et al.^16^ comprehensively and systematically analysed the severity of the consequences of accidents in civil aviation enterprises from 2006 to 2015 using mathematical statistics, revealing the inherent characteristics and patterns of event or accident types and flight periods.

These studies of aviation safety events involve extensive data processing operations, primarily relying on manual processing^17^ or simple visualization of report and chart data using computers^18^, leading to a low processing efficiency, error-prone outcomes, and challenging data quality assurance. With the rise of data-mining technology, it has become possible to extract valuable information and knowledge from large volumes of diverse and complex data^19^.

Data mining originated from various disciplines, with statistics, machine learning, and data warehousing as the most important fields. Friedman^20^ considered data mining as a business information processing technique, revealing hidden, unknown regularities by statistically analysing and predicting vast amounts of data to support decision-making. Therefore, the concept of data mining is often considered equivalent to knowledge discovery in databases (KDD)^21–23^. Currently, data mining, which focuses on accident data analysis using association analysis, cluster analysis, and decision tree analysis algorithms, is mostly applied in fields such as road traffic accidents and coal mine safety^24–29^.

The Apriori algorithm is the first and a classic association rule mining algorithm^30^ and is widely utilized for analysing potential cause-and-effect relationships in maritime shipping accidents, road traffic accidents, and railway incidents. Considerable research in this domain includes studies by Huang et al.^31^, who established a model for analysing association rules in maritime traffic accidents using the Apriori algorithm and proposed strategies to prevent maritime traffic accidents. Yang^32^ utilized the Apriori algorithm to analyse causality in road traffic accidents. Xu et al.^7^ investigated the causality of railway traffic accidents using the Apriori algorithm. ShuangLi et al.^33^ constructed a Bayesian network model through text mining techniques and strong association rules and conducted sensitivity analysis and critical path analysis, thereby elucidating the fundamental causes of mining accidents. Liu et al.^34^ performed a correlation rule analysis of railway operation accidents by improving the Apriori algorithm, extracting 90 causal factors of railway accidents, discovering 159 associated rules, and identifying key causes, interrelated key causes, and accident causation patterns. Li et al.^35^ employed text mining techniques to extract 37 unsafe behaviours and their causative feature words using the Apriori algorithm for association rule mining, ultimately obtaining six core causes and six sets of core associated factors. Li et al.^36^ explored the relationships and interdependence among different causes of building collapse accidents using spectral clustering and the Apriori algorithm, categorizing 43 accident causes into five groups, determining the most crucial cause combinations within each cluster, and proposing targeted measures. Jing et al.^37^ used the Apriori algorithm and Gephi visualization method for statistical analysis of reported data on coal mine accidents across China from 2018 to 2022 to reveal the complex relationships among individual causative factors.

The above literature review reveals that statistical analysis of aircraft accidents has focused mainly on the accident severity, type, month, geographical location and other factors. Text data mining is mainly employed in the railway, maritime accident and construction industries, but it is rarely adopted in the aviation field. To reveal the complex relationships among the causes of aircraft accidents, the Apriori algorithm was used to mine association rules and generate a visual network graph of the obtained association rules.

## Methodology

### Data preprocessing

Data preprocessing involves transforming the original data, correcting errors, deleting redundant data, and modifying inconsistent and incomplete data. Incident investigation reports are typically presented in document form, which is limited by the presentation and content completeness, as well as by the industry knowledge of the investigator. There is no standardized text format, as described in Table 1. Therefore, to ensure the accuracy of the subsequent excavation and analysis, data from accident investigation reports must be preprocessed.

**Table 1:** Items of incident investigation statistics.

Items 1–14	Items 15–28	Items 29–42	Items 43–56
Serial No	Incident Group Serial No	Incident Serial No	Time of Incident
Aircraft Registration No	Location of Occurrence	Title	Initial Fill Unit
Identification	Nature of Flight	Incident Level	Incident Type (Major)
Incident Type 2	Incident Type 3	Incident Cause (Primary)	Incident Cause 2
Incident Cause 3	Primary Responsible Unit	Aircraft 1 Aircraft Type	Aircraft 1 Aircraft Number
Aircraft 1 Using Unit	Aircraft 1 Local Supervisory Authority	Aircraft 1 Local Management Bureau	Responsible Unit 2
Brief History	Event Status	Place of Occurrence	Supervisory Authority at the Place of the Incident
Bureau of Incident Management	Late Reporting or Not	Aircraft 1 Take-Off Site	Aircraft 1 Planned Landing Site
Aircraft 1 Incidence Phase	Aircraft 2 Aircraft Type	Aircraft 2 Aircraft Number	Aircraft 2 Using Unit
Aircraft 2 Local Supervisory Authority	Aircraft 2 Local Management Bureau	Aircraft 2 Incidence Phase	Flight No
Urgent or Not	Occurrence Outside the Country	Related to a Foreign Airline	Statistics or Not
Total Casualties	Total Fatalities	Accountability Status	Cause Analysis
Adopt Measures	Reasons for Late Reporting	Transmission Time	Last Updated

**Figure 3:**
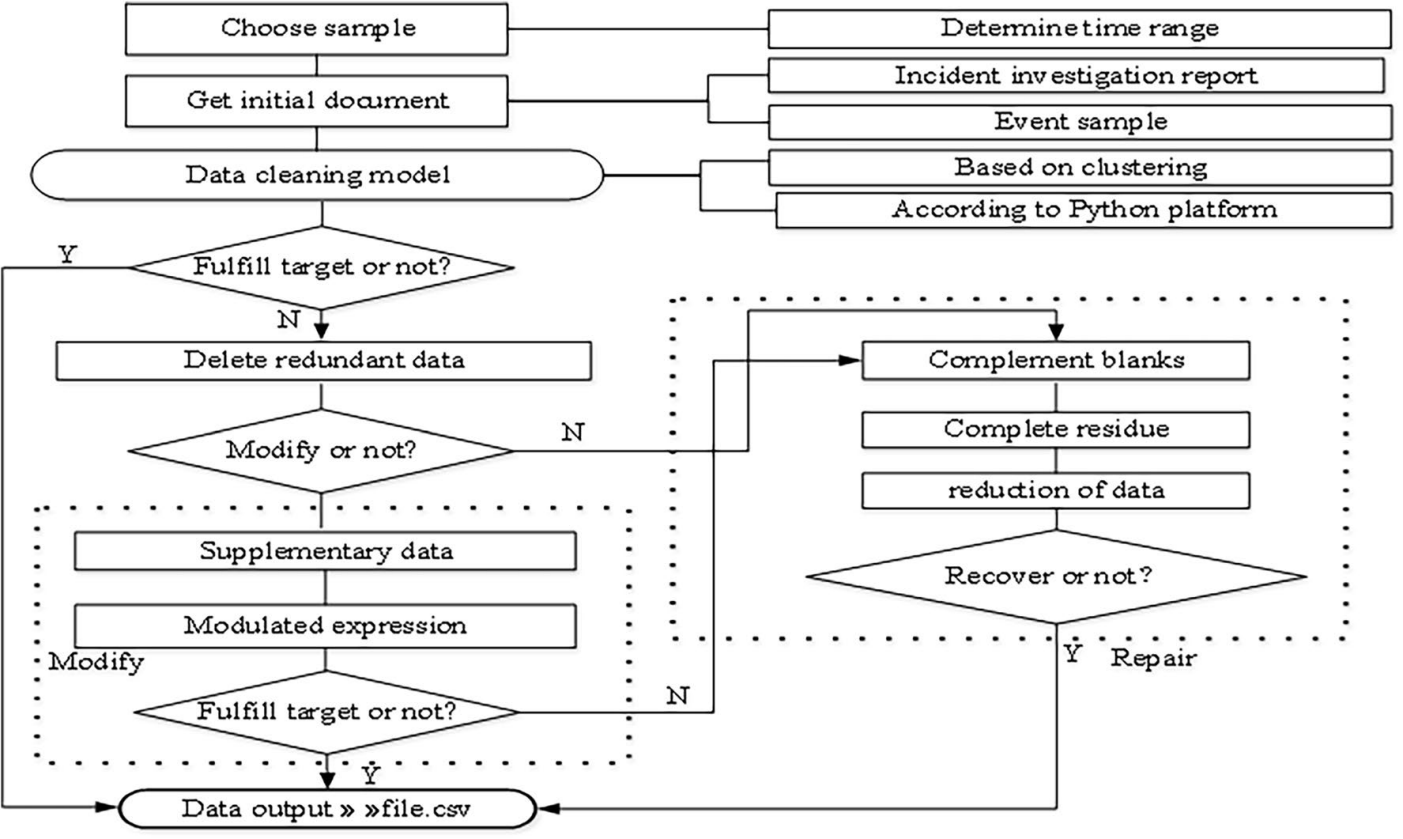
Data cleaning process.

The determination of the causes of aircraft incidents adheres to the principles of completeness, continuity, and consistency. These principles conform with industry regulations and operational manuals. By consulting numerous unsafe event investigation experts and comparing common data cleaning methods, clustering was used to identify outliers by dividing the data of general unsafe events, general incidents and serious incidents into three groups. The 56 retained items are assigned a value of 1, while the deleted items are assigned a value of 0. The specific cleaning process is shown in Fig. 3.

A processed dataset is generated as output in accordance with the data format requirements of the code of the data analysis and processing modules (e.g., numpy, matplotlib, and pandas)

### Feature extraction

The initial feature set obtained after data cleaning based on the expert system model typically consists of high-dimensional data, and not all features are equally important. Irrelevant information can reduce the algorithm performance, leading to dimensionality, ultimately affecting the outcome of data analysis. Introducing feature reduction facilitates the elimination of redundant dimensions (weakly correlated dimensions) or the extraction of more valuable features, thereby increasing the computation speed, enhancing the efficiency, and ensuring the accuracy of data analysis.

The term frequency–inverse document frequency (TF–IDF) method is a classic weighting calculation technique widely used in recent years for data analysis and information processing. The term frequency (TF) represents the frequency or occurrence of a particular keyword within an entire document. The inverse document frequency (IDF) denotes the inverse of the document frequency and is primarily employed to reduce the impact of common words across all documents that minimally influence the document. The TF–IDF model can be expressed as follows:

1 $$tfidf_{i,j} = tf_{i,j} \times idf_{i,j}$$

where $tfidf_{i,j}$ denotes the product of the term frequency $tf_{i,j}$ and the inverse document frequency $idf_{i,j}$. In the TF–IDF method, the weight is directly proportional to the frequency of occurrence of a given feature in a document and inversely proportional to the number of documents containing this feature in the entire corpus. A higher value of $tfidf_{i,j}$ indicates greater importance of the feature word within the text.

The causative factors obtained by feature extraction were sequentially numbered, constituting the current causative factor set $t = \left\{ {t_{1} , t_{2} , \ldots , t_{i} } \right\}$. Simultaneously, the collected incident investigation reports were sequentially numbered, constituting the collection of incident investigation report texts $D = \left\{ {D_{1} , D_{2} ,..., D_{j} } \right\}$.

The TF value can be calculated as:

2 $$tf_{i,j} = n_{i,j} \backslash \sum\nolimits_{k} {n_{k,j} }$$

where the numerator $n_{i,j}$ denotes the occurrence of a given causative factor in incident investigation report $D_{j}$, and the denominator $\sum\nolimits_{k} {n_{k,j} }$ denotes the total count of all causative factors in report $D_{j}$. The resultant $tf_{i,j}$ provides the frequency of a specific feature word.

The IDF value can be obtained as:

3 $$idf_{i} = \log [ \left| D \right|\backslash \left| {\left\{ {j:t_{i} \in d_{j} } \right\}} \right|]$$

where the numerator $\left| D \right|$ denotes the total number of incident investigation reports and the denominator $\left| {\left\{ {j:t_{i} \in d_{j} } \right\}} \right|$ denotes the number of reports containing word causative factor $t_{i}$. If the considered word is absent in the corpus, zero denominator is obtained. Hence, in general, this can be avoided by adding 1 to the denominator, namely, $1 + \left| {\left\{ {j:t_{i} \in d_{j} } \right\}} \right|$.

4 $$idf_{i} = \log [ \left| D \right|\backslash (1 + \left| {\left\{ {j:t_{i} \in d_{j} } \right\}} \right|)]$$

The TF–IDF model can be expressed as follows:

5 $$tfidf_{i,j} = tf_{i,j} \times idf_{i,j} = \frac{{n_{i,j} }}{{\sum\nolimits_{k} {n_{k,j} } }} \times \log \frac{\left| D \right|}{{1 + \left| {\left\{ {j:t_{i} \in d_{j} } \right\}} \right|}}$$

## Determination of the aircraft incident causal factor set

**Figure 4:**
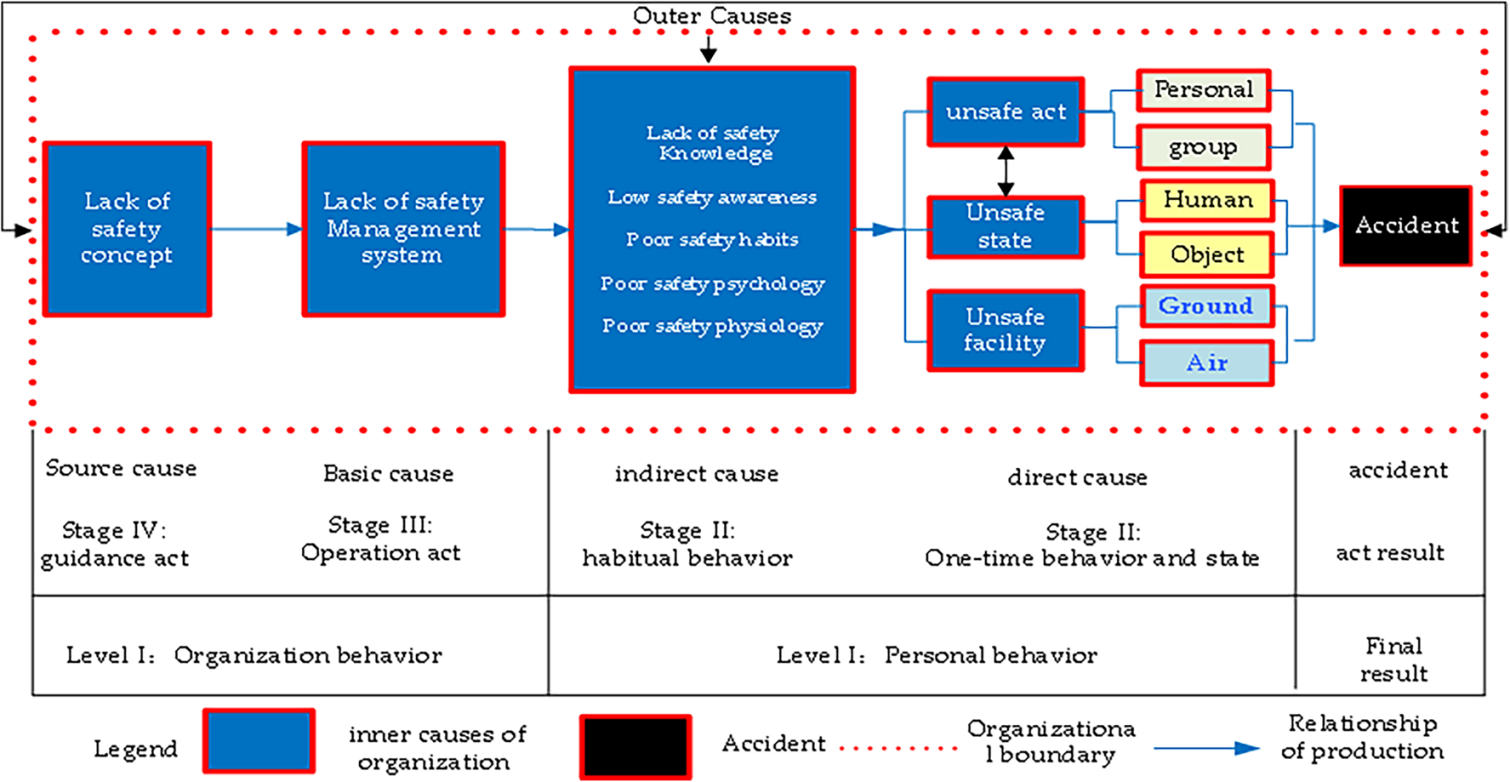
Accident causation 2–4 model.

After data preprocessing, text mining was conducted of the historical aircraft incident investigation reports (Fig. 3). Initially, according to regulations such as the Event Information Reporting and Processing Standard and Event Samples, causative factors were decomposed into relevant causative keywords. With the use of the TF–IDF method for feature word extraction from text, preliminary causative factors of aircraft incidents were identified by matching within specified classified texts. However, the causes of aircraft incidents are multifaceted, and many people are involved. In this paper, the accident causation 2-4 model was introduced. Factors contributing to aircraft incidents in the form of human factors, equipment factors, and management factors were attributed to internal organizational reasons, while the environment was considered an external factor. The specific model is shown in Fig. 4.

The top 30 TF–IDF ranked feature words from each level were selected as causative factors. After deduplication, all the causal factors of each event were obtained. Similar or identical conditions were integrated, and a set of aircraft accident cause factors was finally extracted.

### Aircraft incident causal rule mining

In this paper, the Apriori algorithm of association rules was used to discover relationships or patterns in datasets. Through the downward closure property of frequent item sets, candidate item sets were continuously generated and pruned, and all rules satisfying minimum support and minimum confidence levels were obtained. Association rules that meet the requirements also satisfy the filtering requirements. The greater the support and confidence are, the stronger the rule. In addition, in this paper, the lift index was employed to filter the obtained association rules. A lifting degree higher than 1 indicates that the former and latter terms are positively correlated. Conversely, they are negatively correlated.

### Relevant definitions

The association rule problem based on events can be expressed as follows: let $X = \left( {X_{1} ,X_{2} , \ldots ,X_{m} } \right)$ represent the set of causative factors obtained after data preprocessing, where $m$ is the number of causative factors. $S = \left\{ { S_{1} , S_{2} , \ldots , S_{n} } \right\}$ denotes the original set of association rules for events, with $n$ denoting the total number of association rules, each $S_{i} \left( {1 \le i \le n} \right)$ denoting a subset of item sets, and $S_{i} \in X$.

Event association rules can be expressed as $X_{a} = > X_{b}$, where $X_{a}$ and $X_{b}$ denote the antecedent and consequent, respectively, of the rules. For each rule, $X_{a} \in X$, $X_{b} \in X$ and $X_{a} \cap X_{b} = \emptyset$ must meet the minimum support and confidence thresholds while yielding a lift value greater than 1.

Definition 1 Support: The probability of the simultaneous occurrence of event causative factor items $X_{a}$ and $X_{b}$ is referred to as the event causative rule support, represented by:

6 $$Support\left( { X_{a} = > X_{b} } \right) = {\text{P}}\left( {X_{a} \cap X_{b} } \right) = \frac{{\left| {X_{a} \cup X_{b} } \right|}}{\left| S \right|}$$

where the numerator $\left| {X_{a} \cup X_{b} } \right|$ denotes the count of the simultaneous occurrence of event causative factor items $X_{a}$ and $X_{b}$, and $\left| S \right|$ denotes the total count of all association rules.

Definition 2 Confidence: If an event causative factor item $X_{a}$ occurs, the probability of another event causative factor item $X_{b}$ occurring is referred to as the event causative rule confidence, represented by:

7 $$Confidence\left( { X_{a} = > X_{b} } \right) = {\text{P}}\left( {X_{a} |X_{b} } \right) = \frac{{\left| {X_{a} \cup X_{b} } \right|}}{{\left| {X_{a} } \right|}}$$

where the numerator $\left| {X_{a} } \right|$ denotes the count of association rules containing both causative factor items $X_{a}$ and $X_{b}$, and the denominator $\left| {X_{a} \cup X_{b} } \right|$ denotes the count of association rules containing event causative factor items $X_{a}$ and $X_{b}$.

Definition 3 Lift: The measure of improvement in the probability of the occurrence of one event causative factor item $X_{a}$ in the presence of another event causative factor item $X_{b}$ can be expressed as:

8 $$Lift\left( {X_{a} = > X_{b} } \right) = \frac{{Confidence\left( {X_{a} = > X_{b} } \right)}}{{Support\left( {X_{b} } \right)}}$$

### Antecedents and consequents of the association rule

The core of association rules is to reveal the relationships between items in a dataset, helping to better understand the frequency at which one item set may occur given another. By identifying these relationships, potential patterns and regularities can be determined, providing support for decision-making and prediction. Association rules consist of two parts: the antecedent and the consequent. The antecedent is the condition, while the consequent is the result. The relationship between these parts indicates a trend where certain items may occur in the presence of other items. Association rules were mined based on the support and confidence. The support is a measure of the frequency of simultaneous occurrence of item sets, while the confidence is a measure of the probability of consequent occurrence given the antecedent.

In association rules, the order of the antecedent and consequent is a key concept but can also lead to confusion. Because association rules describe item sets based on their content rather than their order, the order of item sets does not affect the meaning of the rules. In other words, whether the antecedent or consequent, as long as their contents are the same, the meaning of the rules is the same, indicating some form of correlation or causality between two item sets. However, while conceptually, the order of the antecedent and consequent does not affect the meaning of the association rules, it can affect the calculation of metrics such as the support and confidence. This difference occurs because these metrics are calculated based on specific combinations of item sets, reflecting the degree and frequency of association between different item set combinations. Therefore, even if two rules express the same association relationship, their metrics, such as the support and confidence, may differ due to the actual occurrences in the dataset.

In conclusion, the meaning of the obtained association rules depends on the content of their antecedents and consequences, while the metrics reflect the performance and degree of association of these rules in the actual datasets.

### Algorithmic improvements

In the application of the Apriori algorithm for data mining, despite the implementation of conditional checks to avoid division-by-zero errors, not-a-number (NaN) values can still occur. Such instances likely stem from either small data samples or inadequacies in meeting the threshold requirements for metrics such as the support and confidence within certain item sets, thereby resulting in NaN computations. Notably, the emergence of NaN values does not necessarily indicate code errors but may reflect the inherent data characteristics.

In practice, addressing NaN values typically involves employing statistical techniques such as mean or median imputation or adjusting thresholds to mitigate their occurrence. However, due to the distinct nature of aircraft incident data and their difference from conventional datasets, applying statistical methodologies to resolve NaN values, such as using alternative incident cause codes for filling or removing specific cause codes, could alter the interrelations among incident causes, thus compromising the accuracy of the final analysis.

To address this issue, in this paper, an enhancement to the Apriori algorithm was proposed. In contrast to conventional NaN resolution methods, the proposed approach focuses on preprocessing, specifically on filtering and tallying frequent items, aiming to enhance the efficiency and precision of the algorithm. Initially, by traversing each transaction in the dataset, the support of each item can be computed and stored in a header table. Subsequently, items with a support value below the minimum threshold can be removed from the header table, ensuring the retention of only frequent high-support items. Finally, these retained frequent items constitute the item sets. By exclusively considering frequent items, the refined algorithm aims to efficiently extract frequent patterns, thereby augmenting its performance and accuracy.

This preprocessing step reduces the processing time and resource overhead associated with infrequent item handling, consequently lowering the computational complexity and enhancing the algorithmic efficiency and precision. Such a strategy plays a pivotal role in data mining, enabling the algorithm to maintain effectiveness when managing large-scale datasets. Additionally, due to the substantial volume of aircraft incident data employed, an iterative approach was adopted during coding to generate candidate and frequent item sets, avoiding recursive calls and minimizing the recursion depth. This could ensure more effective processing of large datasets while mitigating potential stack overflow issues.

According to the association rule mining method, the steps for mining accident causation association rules are as follows:

Step 1: Input the dataset of accident causation factors.

Step 2: Set the minimum lift threshold, minimum confidence threshold, and minimum support threshold.

Step 3: Utilize the Apriori algorithm for generating strong association rules that meet the minimum support threshold.

Step 4: Filter the obtained frequent item sets based on the minimum support, minimum confidence, and lift thresholds; the rules that meet these criteria are considered association rules.

Step 5: Eliminate association rules where the antecedent or consequent is empty and store the association rules as aircraft incident causal rules.

## Dataset used

### Data collection and cleansing

The dataset utilized in this study was compiled from investigation reports of unsafe events from 2019 to 2022. There are 9835 pieces of data from the Southwest Air Traffic Management Bureau. Due to space limitations, the authors selected only 22 representative data points, which are distributed among different years, different flight stages, different causes and different levels of unsafe aircraft events. The resulting dataset (after data preprocessing) is detailed in Table 2.

**Table 2:** Main investigation attributes after data preprocessing.

Number	Date	Event classification	Causes	Stage
161	2022/12/20	General unsafe event	Altitude Deviation	Climb to Cruise
162	2022/11/20	General unsafe event	Missed Approach	Final Approach
256	2022/11/10	General unsafe event	Missed Approach	Initial Approach
2697	2022/8/26	General unsafe event	Deviation/Mistake SID	Initial Climb
2980	2022/8/18	General unsafe event	Missed Approach	Final Approach
3302	2022/8/8	General unsafe event	Less than Interval	Descent
3471	2022/8/3	General unsafe event	Less than Interval	Cruise
3950	2022/7/21	General unsafe event	Runway Incursion/Occupation	Take-Off
5038	2021/6/13	General unsafe event	Swerve/Slip off the Taxiway	Taxiing
5064	2021/6/12	General unsafe event	Missed Approach	Final Approach
5289	2021/6/4	General unsafe event	Less than Interval	Final Approach
5649	2021/5/22	General unsafe event	Missed Approach	Final Approach
6040	2021/5/11	General unsafe event	ACAS (TCAS) Warning	Descend
6526	2021/4/26	General unsafe event	Less than Interval	Cruise
6775	2021/4/18	General incident	Less than Interval	Descent
6815	2021/4/17	General unsafe event	Altitude Deviation	Descent
7577	2020/3/24	General unsafe event	Less than Interval	Descent
7832	2020/3/15	Serious incident	Runway Incursion/Occupation	Final Approach
8010	2020/3/10	General unsafe event	Missed Approach	Miss Approach
8030	2020/3/8	General unsafe event	Trek/Yaw	Final Approach
8755	2019/2/2	General unsafe event	Proximity Control Interval	Climb to Cruise
8766	2019/2/1	General unsafe event	Other	Taxi to Runway
8972	2019/1/22	General unsafe event	Stop Take-Off	Stop Take-Off

Text mining was applied to the collected event investigation reports. Following existing regulations such as the Event Information Reporting and Processing Standard and Event Samples, causative factors were decomposed into relevant causative keywords. The TF–IDF method was employed for text feature extraction, and preliminary aircraft incident causative factors were obtained by matching within specified classified texts. The accident causation 2-4 model was applied for further screening of the aircraft incident causative factors, categorizing all factors during aircraft operation into human, equipment, management, and environmental layers. Finally, the top 30 TF–IDF-based ranked feature words from each layer were extracted as the causative factors for that layer.

### Encoding of the causal factor set

The obtained 56 items are all data sources from the unsafe incident investigation reports listed in Table 1. After data cleaning with the expert system and factor screening by the accident cause 2-4 model, the 4th, 5th, 6th, 8th, 11th, 14th, 18th, 19th, 20th, 24th, 31st, 32th, 34th, 45th, 46th, 48th, 49th, 50th, 52nd and 55th items were selected. These items include the unsafe event type and all types of causes, including relevant personnel, aircraft, equipment conditions, management and environment. The set of causative factors of aircraft incidents was extracted and encoded, as summarized in Table 3.

**Table 3:** Codes of the causes of aircraft incidents.

Items	Causal Factor Encoding
Human Layer	Crew *H*_01_, Controller *H*_02_, Duty manager *H*_03_, Improper energy allocation *H*_04_, Mishearing *H*_05_, Failure to rectify incorrect recitation *H*_06_, Lack of preparation for unlandable situations* H*_07_, Shift in work focus *H*_08_, Lack of conflict judgement *H*_09_, Poor conflict resolution ability *H*_10_, Inadequate monitoring *H*_11,_ Occurrence of luck mentality *H*_12_, Failure to detect flight conflicts in advance *H*_13_, Scattered work style *H*_14_, Weak safety awareness *H*_15_, Unauthorized departure during duty *H*_16_, Cumulative risk of personnel fatigue *H*_17_, Violation of operational manual regulations *H*_18_, Some regulations were orally requested but not included in the manual *H*_19_, Weak team cooperation awareness *H*_20_, Inadequate risk assessment *H*_21_, Insufficiently stringent work procedures *H*_22_, Inadequate risk identification *H*_23_, Inadequate ability to manage complex situations *H*_24_, Plans did not fully consider the interference caused by sudden situations *H*_25_, Occurrence of inertia thinking *H*_26_, Delayed issuance of landing permits *H*_27_, Inadequate radar monitoring *H*_28_, Insensitivity to alarms *H*_29_, Inadequate onsite management duties *H*_30_, Failure to conduct radar identification as required for aircraft *H*_31_, Insufficient understanding of "highlight display" and "conflict line" significance *H*_32_, Inadequate recognition of key risks in hotspots *H*_33_, Poor work status *H*_34_, Inadequate emergency management *H*_35_, Inconsistent situational awareness *H*_36_, Inadequate mastery of specific content during flight training *H*_37_, Failure to request ascent height in time *H*_38_, Inadaptability to new work procedures *H*_39_, Inadequate technical prevention measures *H*_40_, Weak risk awareness *H*_41_, Excessive delegation of authority* H*_42_, Inadequate estimation of weather impacts *H*_43_, Unclear basic concepts *H*_44_, Inadequate coping ability *H*_45_, Inaccurate language in voice clearance communications *H*_46_, Failure to comply with instructions *H*_47_, Intercepting incorrect flight paths *H*_48_, Inadequate attention to airborne situations *H*_49_
Management Layer	Inadequate rigorous risk control measures* M*_01_, Insufficiently detailed conflict resolution training *M*_02_, Some individuals adopt a sense of luck regarding information reporting *M*_03_, Insufficient safety management *M*_04_*,* Lack of effective supervision and evaluation mechanisms *M*_05_, Inadequate onsite management precision *M*_06_, Inadequate grasp of ideological dynamics in a timely manner *M*_07_, Insufficient differentiated training *M*_08_, Ineffective communication between relevant units *M*_09_, Ineffective work style of management personnel *M*_10_, Inadequate analysis and research of risks *M*_11_, Inadequate daily education of controllers *M*_12_, Inadequate implementation of responsibilities *M*_13_, Inadequate manual promotion *M*_14_, Inconsistent understanding of important regulations *M*_15_, Lack of strict daily supervision *M*_16_, Inadequate depth in case analysis *M*_17_, Inadequate safety pressure transmission *M*_18_, Failure to maintain relevant data as per agreement *M*_19_, Inadequate training *M*_20_, Inadequate regulations *M*_21_, Inadequate combination *M*_22_, Existing loopholes in implementing relevant requirements *M*_23_, Lack of rigorous work processes *M*_24_, Inadequate personnel qualification management M_25_, Inadequate management of team resources *M*_26_
Equipment Layer	Existing technical prevention measures failed to control risks *E*_01_, Meteorological radar equipment did not respond to airborne weather conditions in real time *E*_02_, Lack of alarm prompts when the automated system encounters abnormalities* E*_03_, Lack of equipment support* E*_04_, Difference between permission heights displayed in standby and primary systems *E*_05_, Occurrence of equipment failure during primary and secondary automated system switching *E*_06_, Sudden malfunction of instrument landing system equipment *E*_07_
Environment Layer	Noisy operational environment *S*_01_, Delay in specific transfer of flight information from the previous control unit *S*_02_, Momentary busy period of the tower control channel *S*_03_, Nonstop flight construction *S*_04_, Low-level flight volume leading to relaxation *S*_05_, Scattered thunderstorm weather conditions *S*_06_, Presence of air force activities *S*_07_, Sudden isolated thunderstorm weather conditions *S*_08_
Event Type	General unsafe event *A*_01_, General incident *A*_02_, Serious incident *A*_03_

### Unsupervised learning of causal analysis

**Figure 5:**
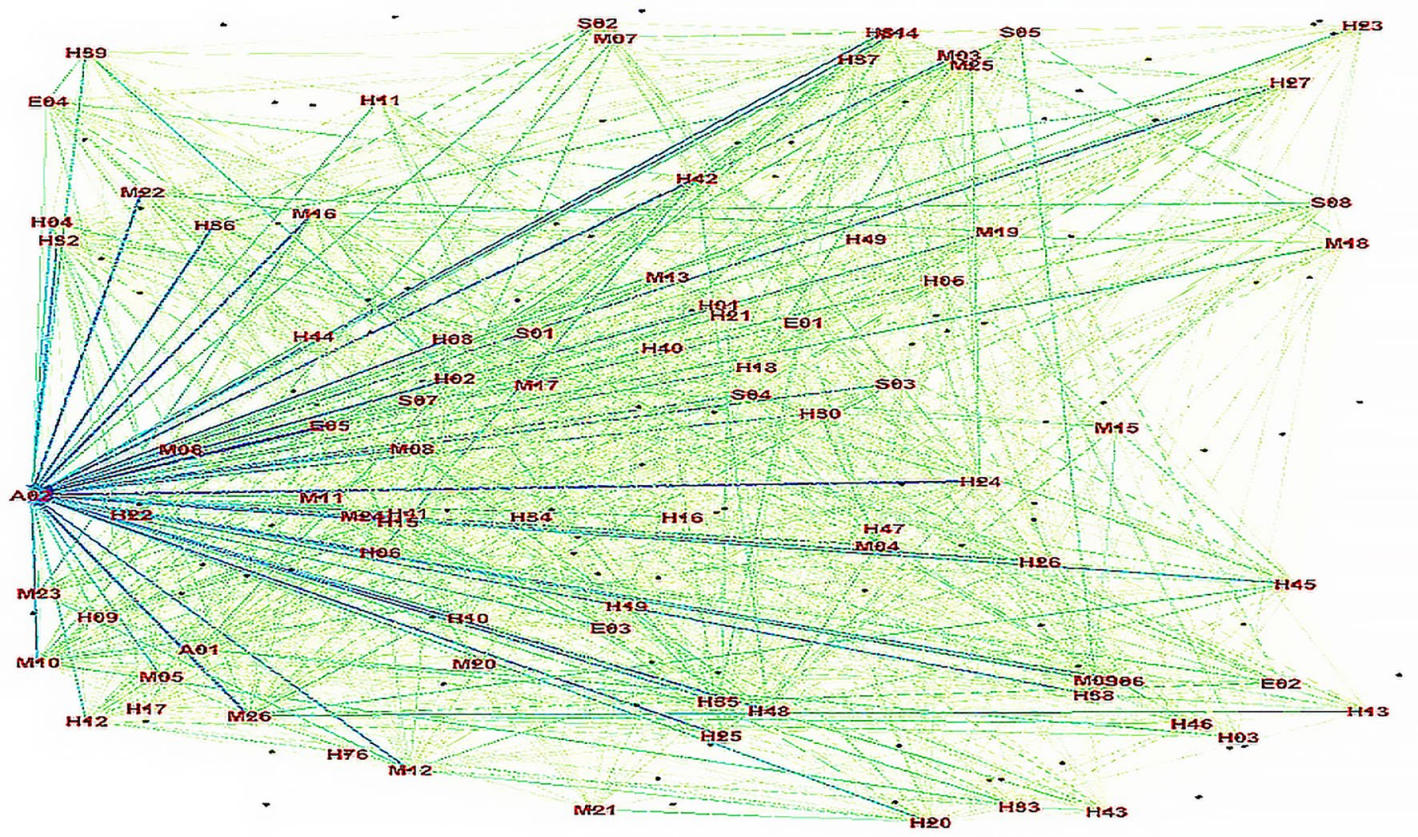
Initial association network of the causal relationships from unsupervised learning.

The set of causal factors used to train the machine model lacks labels, requiring it to autonomously explore, obtain, and summarize knowledge to annotate the training data. This facilitates the discovery of inherent patterns and features among these elements. In this study, an initial correlation network for aircraft accident causation was established, as shown in Fig. 5.

**Figure 6:**
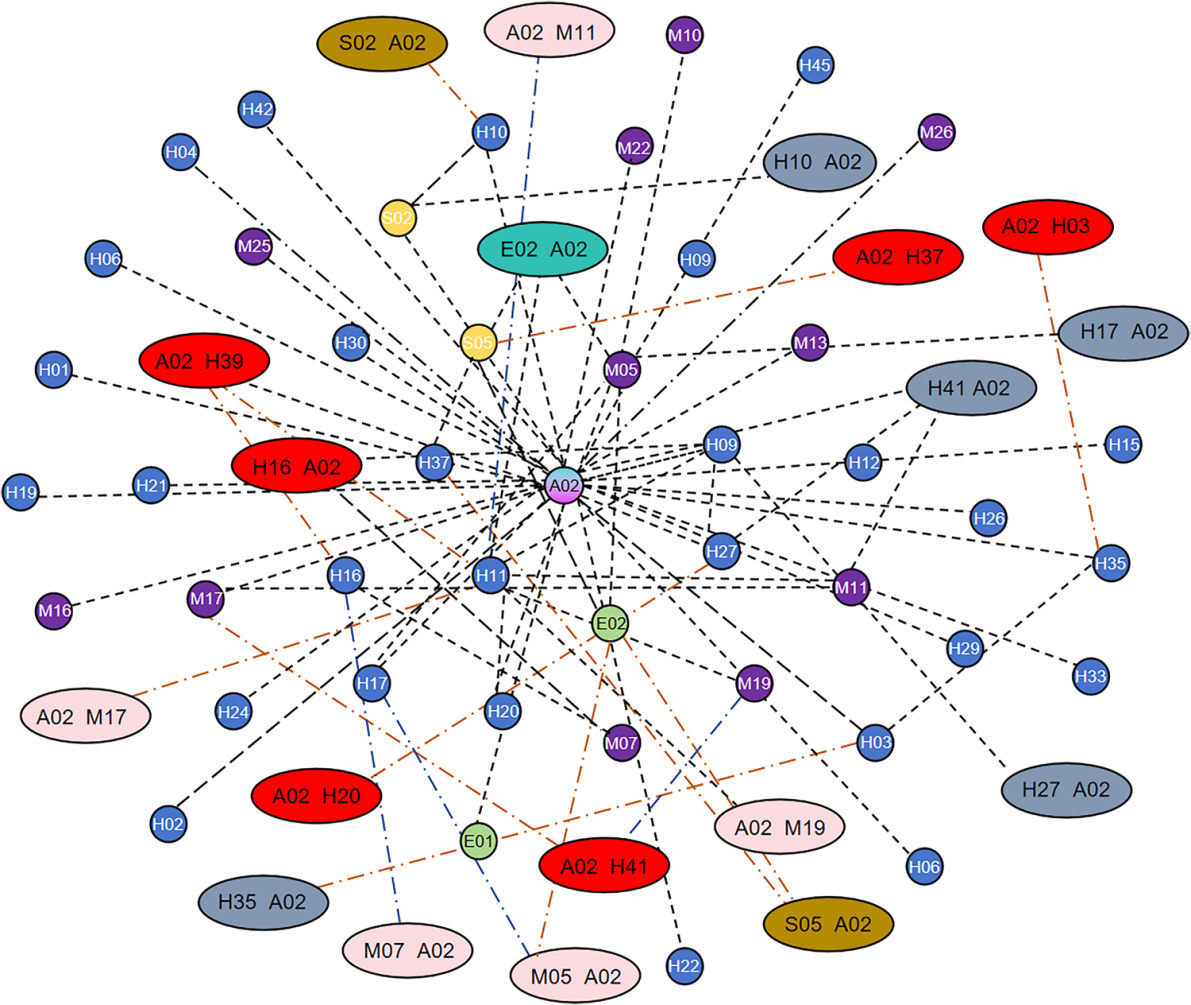
Network graph of the discovered association rules.

Figure 5 clearly shows that node *A*_02_ occupies a central position and exhibits connections with numerous factors. However, due to the considerable number of nodes and edges, deriving precise connections between causal factors remains challenging, thereby hindering quantitative analysis of their relationships. To reveal valuable yet hidden associations, the subsequent step involved employing the Apriori algorithm for data mining. This approach aimed to reveal the latent value within the dataset, resulting in the determination of association rules meeting specific conditions. The network graph of these rules is shown in Fig. 6.

**Figure 7:**
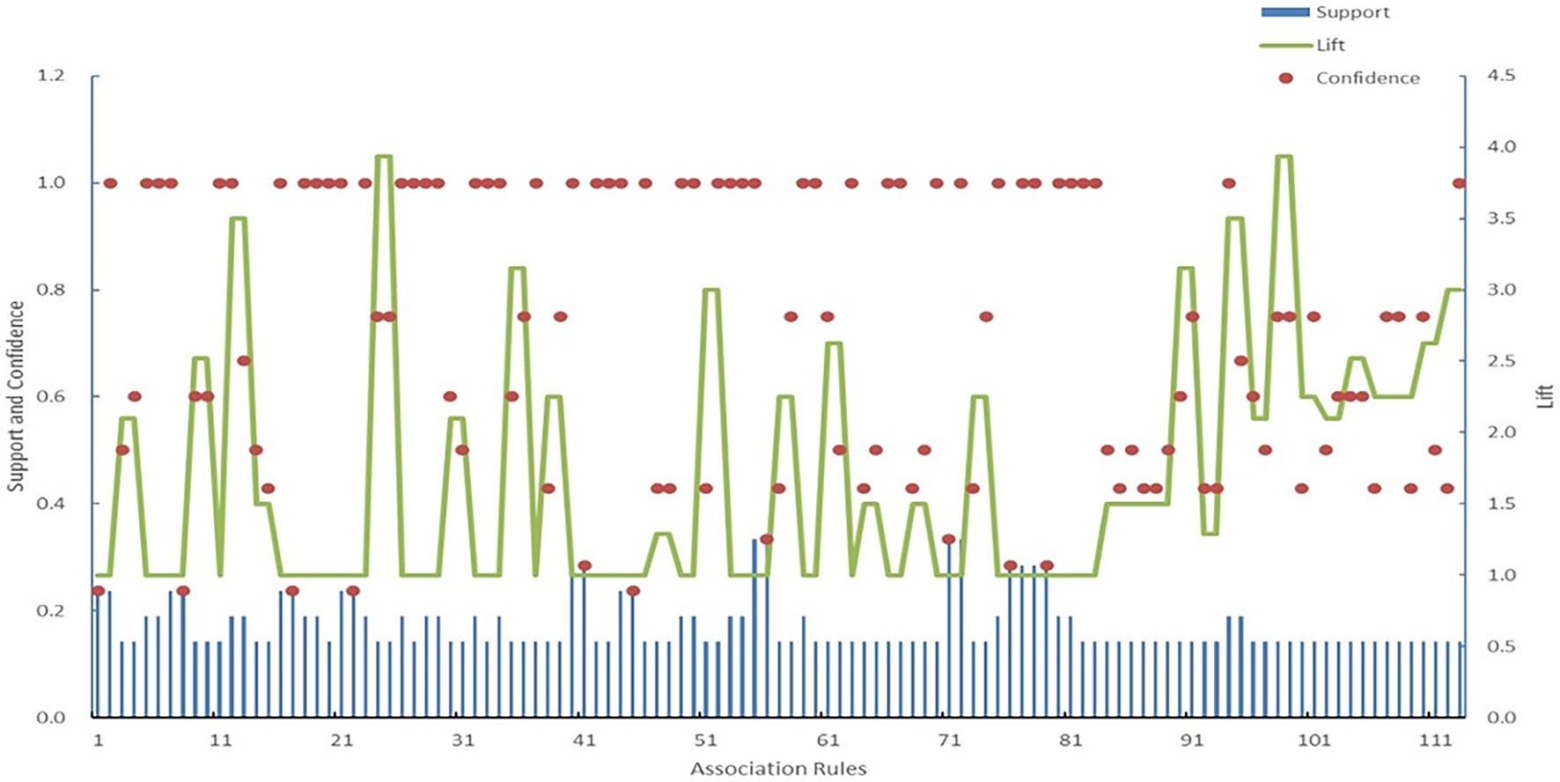
Scatter plot of the support, confidence and lift from unsupervised learning.

In Fig. 6, node *A*02 remains at the network centre, demonstrating connections with multiple nodes such as *M*05, *E*02, and *H*67, among others. Nevertheless, it remains challenging to quantitatively analyse these association rules. Therefore, the introduction of quantitative evaluation through the support, confidence, and lift was necessary, as shown in Fig. 7.

An analysis of the support depicted in Fig. 7 reveals frequently occurring risk factors in accidents, indicating their propensity to cause the risk state of an aircraft incident. Moreover, the analysis of high-confidence association rules reflects reliable cause–effect relationships. Association rules with high lift indicate positive or negative combinations of factors. However, in the scatter plot, numerous association rules exhibit a confidence level of 1, suggesting a high-confidence association between factors. Some feature value pairs are detailed in Table 4.

**Table 4:** Some of the high-confidence association rules from unsupervised learning.

No	Antecedent	Consequent	Support	Confidence	Lift
1	H37	A02	0.238095238	1	1
2	M25	A02	0.19047619	1	1
3	H10	A02	0.19047619	1	1
4	M07	A02	0.238095238	1	1
5	H19	A02	0.142857143	1	1
6	H17	M05	0.19047619	1	3.5
7	H24	A02	0.238095238	1	1
8	E01	A02	0.19047619	1	1
9	H12	A02	0.19047619	1	1
10	H42	A02	0.142857143	1	1

**Figure 8:**
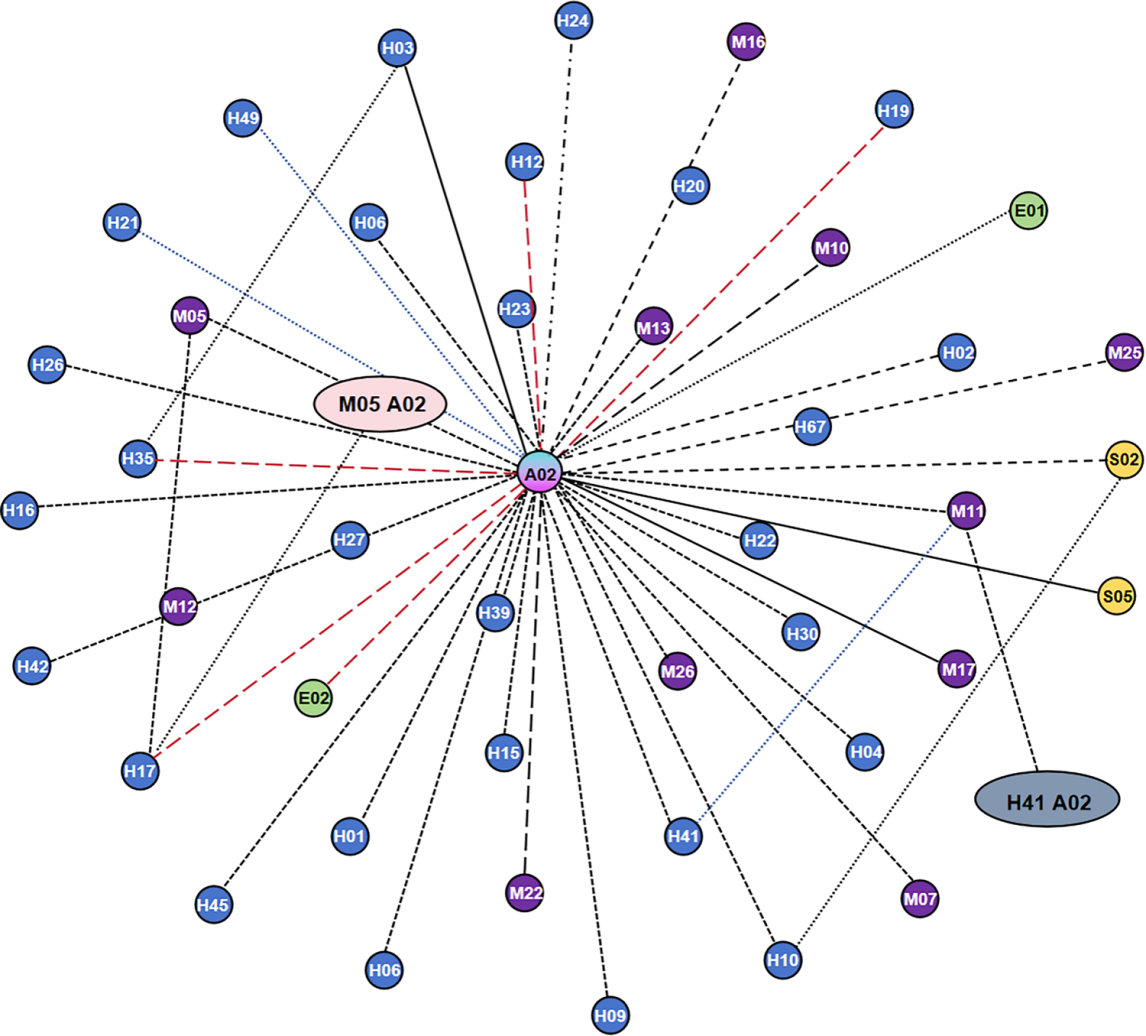
High-confidence network graph from unsupervised learning.

Table 4, which is based on the definition of confidence, indicates that inadequate mastery of specific content during flight training *H*_37_, inadequate personnel qualification management *M*_*25*_, and poor conflict resolution ability *H*_10_ are likely to cause the occurrence of a general incident *A*_02_. To more comprehensively visualize the association rules causing the occurrence of a general incident *A*_02_, a high-confidence network graph was generated, as shown in Fig. 8.

Figure 8 shows that *A*_02_ remained at the network centre and was interconnected with multiple nodes, indicating that many factors potentially cause *A*_02_. However, the association rules are overly idealized, relying too heavily on individual factors while disregarding other factors that might contribute to its occurrence, thereby potentially impacting the accuracy of the final analysis.

### Causal analysis using supervised learning

#### Initial association

**Figure 9:**
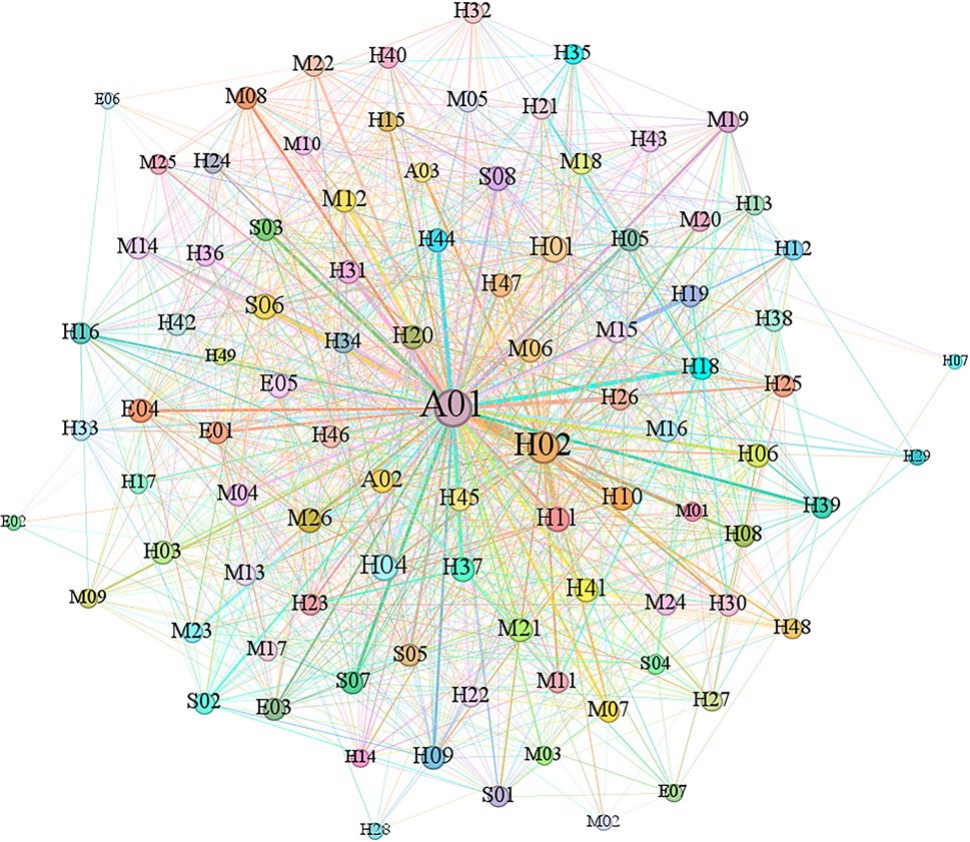
Incident cause correlation network from supervised learning.

To enhance the analysis accuracy, supervised learning was applied to prelabel the original training set, thereby adjusting or removing association rules with a confidence value of 1. A new set of associations was then established after this step, leading to changes in confidence values. The resulting causal correlation network of aircraft incident causes after data intervention is shown in Fig. 9. In the causality network graph, each node represents the causal factors extracted from the selected practice survey reports, including 90 causal factors and 3 incident types, and the node size is determined by the degree of the node. The correlation between the causal factors is regarded as an undirected edge between the nodes; if 2 causal factors occur in one event at the same time, there is an edge between the two points, and the weight of the edge is the number of accidents in which both factors concurrently appear.

Figure 9 shows that *A*_01_ and *H*_02_ are central nodes in the network graph. In contrast to the unsupervised algorithm results shown in Fig. 5, where only *A*_02_ emerged as the central node, this revised network graph more explicitly highlights the significance of controllers in safeguarding against aircraft incidents. Through supervised learning intervention, extreme situations in the association rules of the original training set could be addressed, allowing the analysis of association rules between factors to expand beyond the connections of a single node with others.

The Apriori algorithm was utilized to mine association rules while adjusting the minimum support, confidence, and lift thresholds. Different minimum support thresholds yielded varying quantities of association rules, as detailed in Table 5.

**Table 5:** Changes in the association rules for different minimum support thresholds.

min_support = 0.09					
	Antecedent	Consequent	Support	Confidence	Lift
0	M05	E01	0.095238	0.333333	1.75
1	E01	M05	0.095238	0.500000	1.75
2	H41	H04	0.095238	0.285714	1.50
…	…	…	…	…	…
1182	S05	A02, M26, M07, H16, H04	0.095238	0.333333	3.50
1183	H16	A02, M26, M07, S05, H04	0.095238	0.400000	4.20
1184	H04	A02, M26, M07, S05, H16	0.095238	0.500000	5.25
min_support = 0.2					
0	A02	H35	0.238095	0.238095	1.0
1	H35	A02	0.238095	1.000000	1.0
2	H24	A02	0.238095	1.000000	1.0
…	…	…	…	…	1.0
17	M05	A02	0.285714	1.000000	1.0
18	M07	A02	0.238095	1.000000	1.0
19	A02	M07	0.238095	0.238095	1.0
min_support = 0.05					
0	A01	H11	0.057143	0.142857	1.666667
1	H11	A01	0.057143	0.666667	1.666667
2	H21	A02	0.057143	0.666667	2.333333
…	…	…	…	…	…
125	M06	M14, A03	0.057143	0.400000	4.666667
126	M14	M06, A03	0.057143	0.500000	8.750000
127	A03	M06, M14	0.057143	0.200000	3.500000

**Figure 10:**
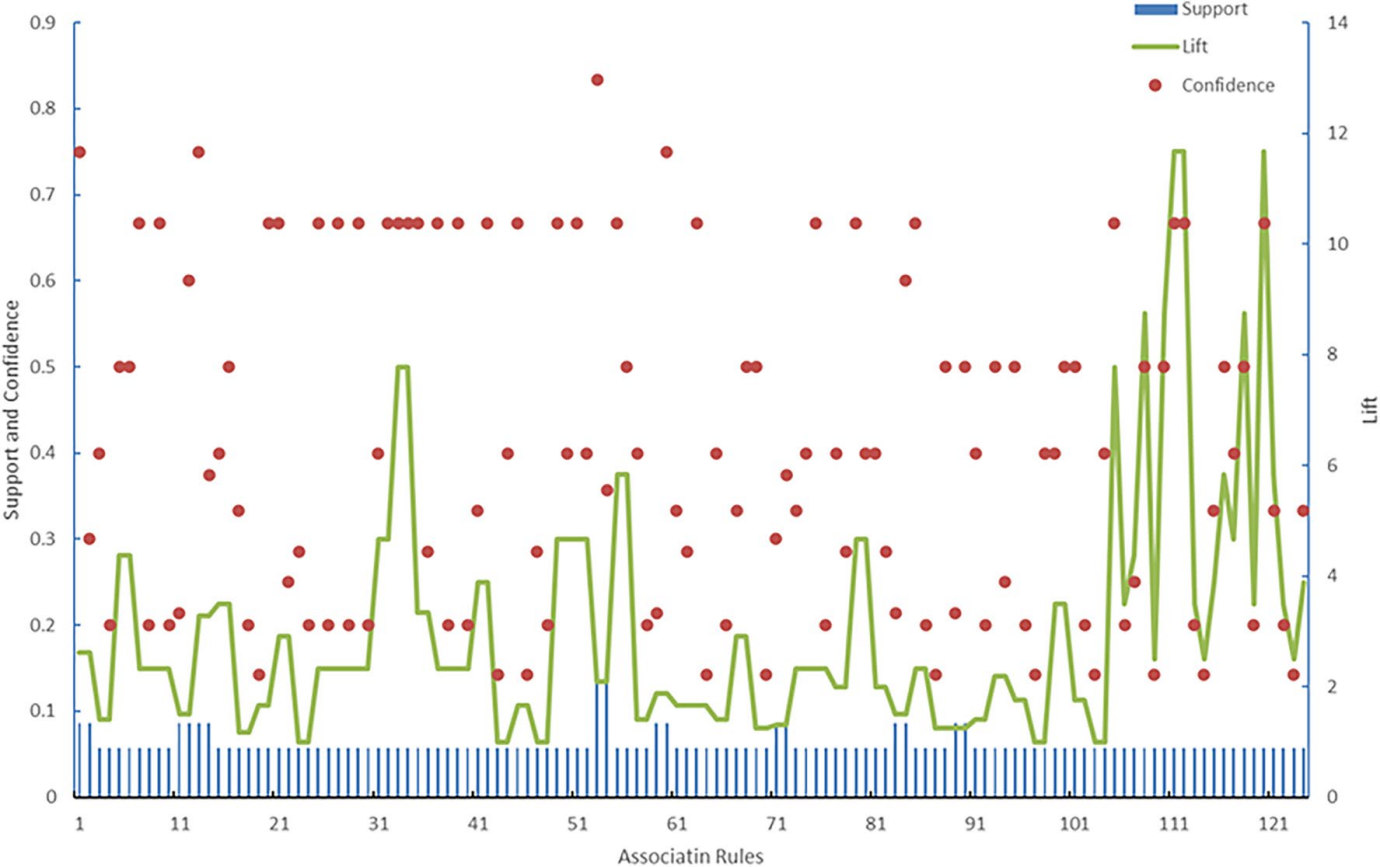
Scatter plot of the support, confidence, and lift of the association rules from supervised learning.

To ensure the analysis accuracy, thresholds were set to filter out low-reliability association rules while obtaining a sufficient quantity for analysis. Thus, by setting the minimum support, confidence, and lift thresholds to 0.05, 0.1, and 1, respectively, rule mining was performed. After data mining, 128 association rules were obtained. A scatter plot of their support, confidence, and lift is shown in Fig. 10.

**Figure 11:**
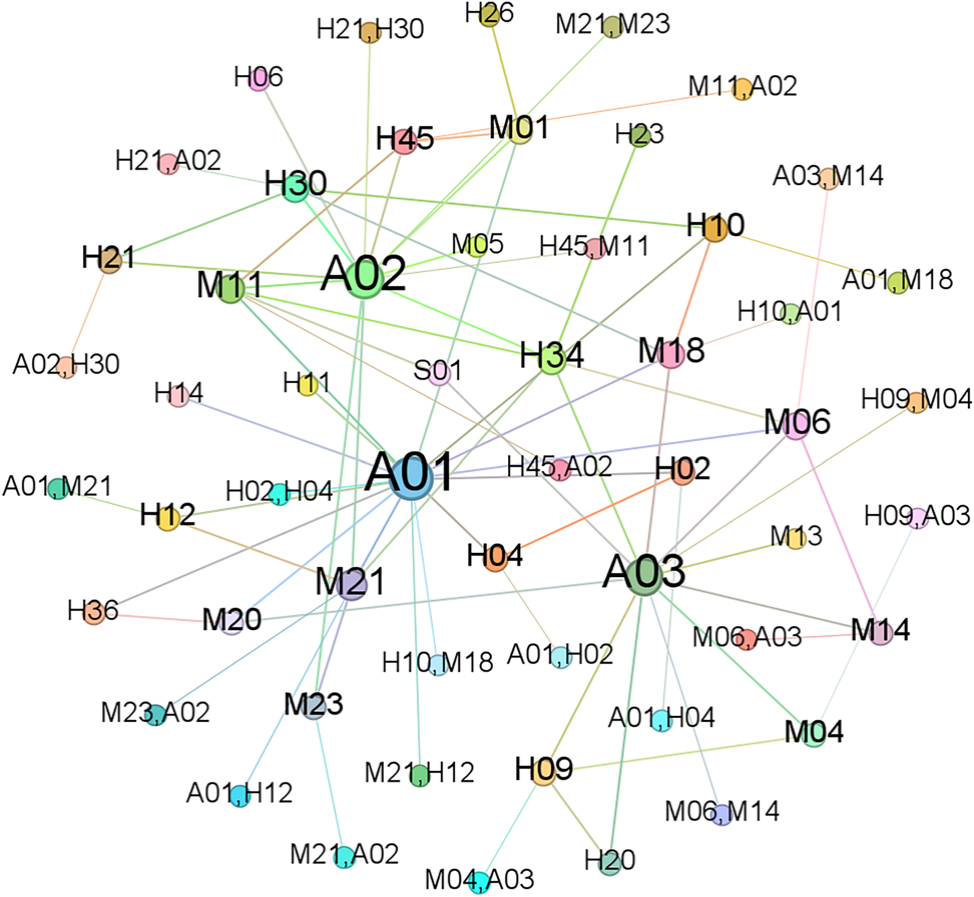
Diagram of the 128 association rules.

Figure 11 shows that certain nodes occur at the centre of the network graph, exhibiting more complex connections with other nodes. These nodes include general incident *A*_02_, serious incident *A*_03_, inadequate safety pressure transmission *M*_18_, insufficient regulation *M*_21_, inadequate onsite management duties *H*_30_, inadequate regulation *M*_21_, inadequate coping ability *H*_45_, and inadequate rigorous risk control measures *M*_01_. These nodes exhibit higher degrees than other nodes, indicating more frequent association rules with other nodes. This emphasizes the need for specific attention and strict control of these nodes in civil aviation safety management.

Based on the diagram, it can be preliminarily determined that *A*_01_, *A*_02_ and *A*_03_ hold central positions in the network graph and possess significant weights. In contrast to the unsupervised algorithm results in Fig. 6, despite containing fewer nodes, the variations among different association rule indicators increased. This approach is more advantageous for subsequent analyses of the relationships between association rules, enhancing the credibility of the analysis results.

#### Analysis of the high-support association rules

Fifty association rules with high support were extracted from all association rules. These high-support association rules are detailed in Table 6.

**Table 6:** Some of the high-support association rules from supervised learning.

No	Antecedent	Consequent	Support	Confidence	Lift
1	H02	A01	0.142857143	0.833333333	2.083333333
2	A01	H02	0.142857143	0.357142857	2.083333333
3	M14	A03	0.085714286	0.75	2.625
4	A03	M14	0.085714286	0.3	2.625
5	A01	H04	0.085714286	0.214285714	1.5
6	H04	A01	0.085714286	0.6	1.5
7	H10	H30	0.085714286	0.75	3.28125
8	H30	H10	0.085714286	0.375	3.28125
9	A01	H12	0.085714286	0.214285714	1.875
10	H12	A01	0.085714286	0.75	1.875

**Figure 12:**
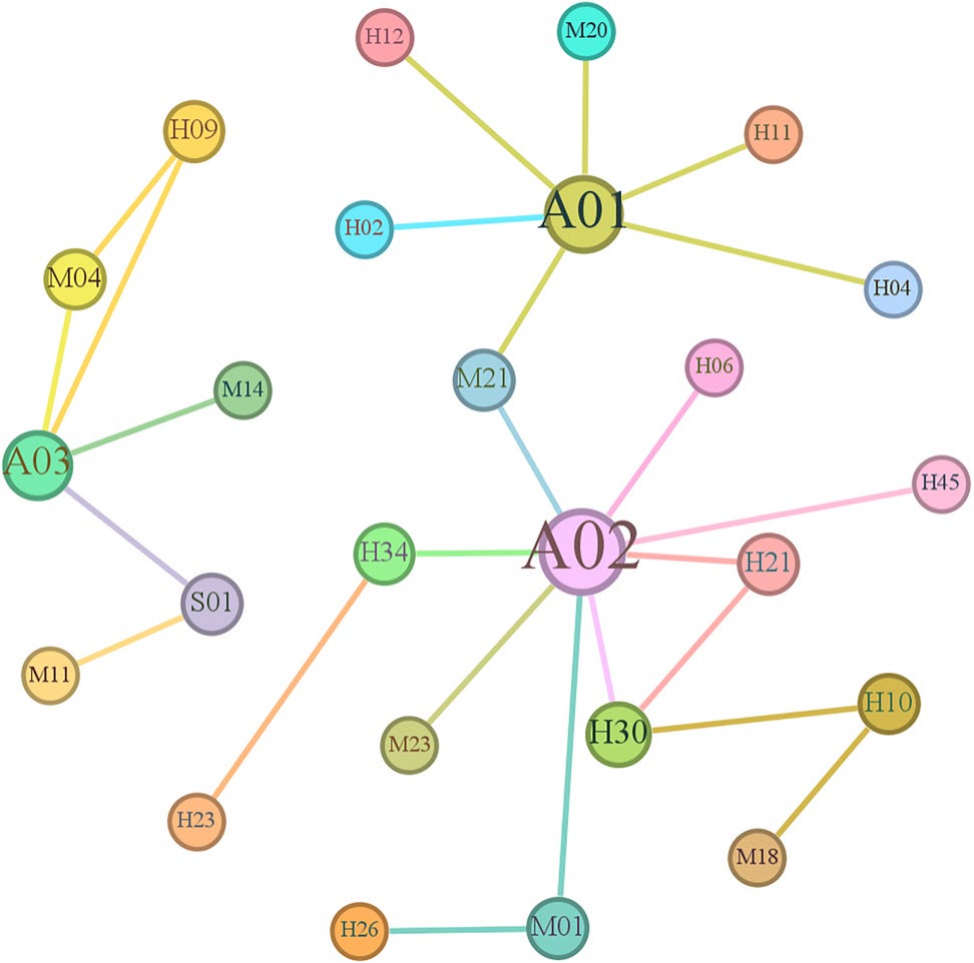
Diagram of the high-support association rules.

The 50 high-support association rules exhibited support values varying between 0.057142 and 0.142858, confidence values varying between 0.2 and 0.833334, and lift values varying between 1 and 4.375. High-support association rules indicate frequent relationships between factors, with higher support indicating stronger rules. A diagram of the high-support association rules is shown in Fig. 12.

The analysis revealed important nodes, such as *A*_01_, *A*_02_, *A*_03_, *H*_30_, *M*_21_, and *H*_10_. The frequent relationships between the identified factors include correlations between air traffic controllers *H*_02_ and general unsafe events *A*_01_, between inadequate manual promotion *M*_14_ and serious incidents *A*_03_, between improper energy allocation *H*_04_ and general unsafe events *A*_01_, and between poor conflict resolution ability *H*_10_, inadequate onsite management duties* H*_30_, and occurrence of luck mentality *H*_12_ and general unsafe events *A*_01_. These frequent influences between factors contribute to the aircraft operational system occurring in a high-risk state, leading to aircraft incidents. Hence, focused attention and preventive measures are needed for the corresponding personnel, equipment, management, and environmental factors related to these causal factors to minimize their impact.

#### Analysis of the high-confidence association rules

Fifty association rules with high confidence were selected from the 128 association rules. These high-confidence association rules are detailed in Table 7.

**Table 7:** Some of the high-confidence association rules from supervised learning.

No	Antecedent	Consequent	Support	Confidence	Lift
1	H02	A01	0.142857143	0.833333333	2.083333333
2	M14	A03	0.085714286	0.75	2.625
3	H10	H30	0.085714286	0.75	3.28125
4	H12	A01	0.085714286	0.75	1.875
5	M23	A02	0.057142857	0.666666667	2.333333333
6	H06	A02	0.057142857	0.666666667	2.333333333
7	H11	A01	0.057142857	0.666666667	1.666666667
8	H21	H30	0.057142857	0.666666667	2.916666667
9	H09	A03	0.057142857	0.666666667	2.333333333
10	H45	A02	0.057142857	0.666666667	2.333333333

**Figure 13:**
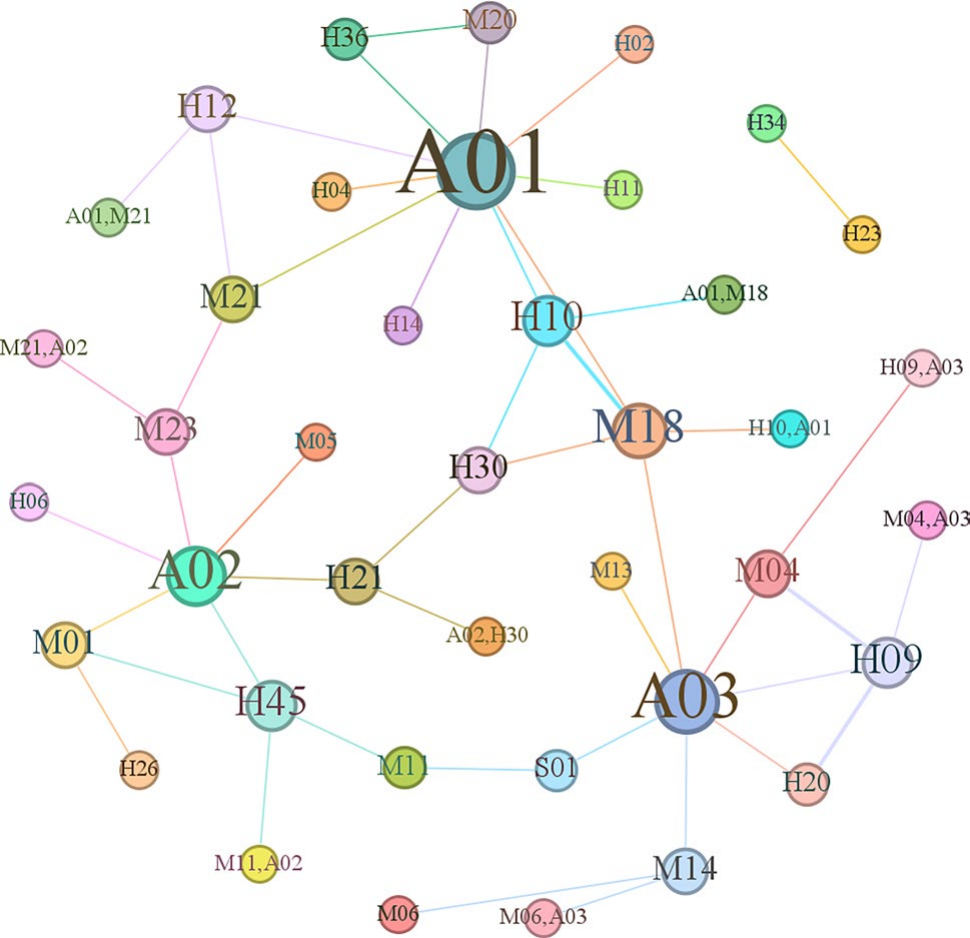
Diagram of the high-confidence association rules.

These 50 high-confidence association rules exhibited confidence values varying between 0.5 and 0.833334, support values varying between 0.057142 and 0.142858, and lift values varying between 1.25 and 11.666667. Analysis of the high-confidence association rules, represented in the network diagram shown in Fig. 13, provides more intuitive judgement of the relationships between factors.

An analysis of the diagram reveals that the occurrence of aircraft general unsafe events *A*_01_ is highly likely due to controller oversight *H*_02_. There is a 75% chance of personnel-related factors such as the occurrence of luck mentality *H*_12_ causing the occurrence of aircraft general unsafe events *A*_01_ during work. Similarly, serious incidents *A*_03_ are 75% likely to occur due to inadequate manual promotion *M*_14_. When a general incident *A*_02_ occurs, there is a 66.7% likelihood of it being caused by a failure to rectify incorrect recitation *H*_06_. Such high-confidence association rules highlight significant causal relationships, indicating that certain antecedent factors are highly likely to cause subsequent factors, thereby increasing the risk of aircraft incidents.

#### Analysis of the high-lift association rules

Fifty association rules with high lift were selected from the 128 association rules. Some of these high-lift association rules are detailed in Table 8.

**Table 8:** Some of the high-lift association rules from supervised learning.

No	Antecedent	Consequent	Support	Confidence	Lift
1	H09	M04,A03	0.057142857	0.666666667	11.66666667
2	M04	H09,A03	0.057142857	0.666666667	11.66666667
3	M23	M21,A02	0.057142857	0.666666667	11.66666667
4	H45	M11,A02	0.057142857	0.666666667	11.66666667
5	H10	A01,M18	0.057142857	0.5	8.75
6	M18	H10,A01	0.057142857	0.5	8.75
7	M14	M06,A03	0.057142857	0.5	8.75
8	H09	M04	0.057142857	0.666666667	7.777777778
9	M04	H09	0.057142857	0.666666667	7.777777778
10	H21	A02,H30	0.057142857	0.666666667	7.777777778

**Figure 14:**
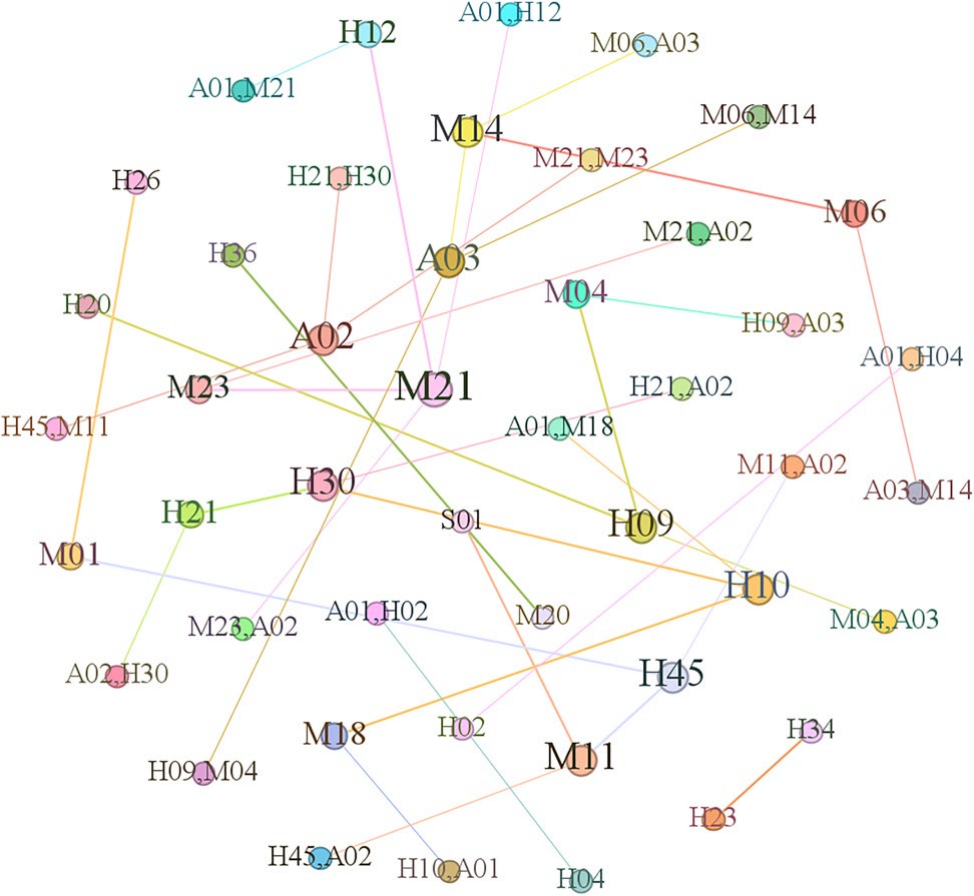
Diagram of the high-lift association rules.

These 50 high-lift association rules exhibited lift values varying between 2.625 and 11.666667, support values varying between 0.057142 and 0.085715, and confidence values varying between 0.2 and 0.75. The network diagram shown in Fig. 14, which was created based on these high-lift association rules, illustrates the relationships between factors more explicitly.

The analysis indicates that the highest lift is observed between factors such as lack of conflict judgement *H*_09_, insufficient safety management *M*_04_, and serious incidents *A*_03_, suggesting a strong positive correlation among these three factors. This suggests that the occurrence of a lack of conflict judgement *H*_09_ and insufficient safety management *M*_04_ might increase the risk of serious incidents *A*_03_, ultimately leading to their occurrence.

## Conclusion and prospects

The current practice of aviation event prediction often fails to capitalize on valuable insights from historical data, primarily focusing on immediate operational parameters. This study focused on addressing this gap by leveraging historical aviation event data and data mining techniques, in accordance with relevant regulations, to extract insights, analyse causal relationships, and enhance operational safety.

We utilized the TF–IDF technique for feature extraction and identified ninety causal factors based on the event causality 2-4 model. These factors were categorized into four layers: human (49 factors), equipment (8 factors), management (26 factors), and environment (7 factors) layers.

The Apriori algorithm was modified to mine association rules from frequent patterns in an unsupervised learning framework, compensating for the absence of date labels. Network graph analysis was employed to initially identify associations between the data, aiding feature extraction. However, early results indicated a prevalence of association rules with a confidence level of 1, suggesting overly idealized associations with low causal analysis accuracy. To resolve this issue, supervised learning techniques were applied to adjust and refine these rules, with continual calibration of the support and confidence thresholds initially set to 0.05 and 0.1, respectively. This process yielded 128 meaningful association rules, which were analysed for their support, confidence, and lift to quantify the underlying associations.

In this study, aviation event influencing factors were comprehensively analysed from multiple perspectives and levels, uncovering potential patterns and characteristics of mutual interactions among various types of historical event data. By quantitatively analysing the relationships among aviation event factors, the reliability of aviation event analysis can be enhanced.

According to the exploration of historical aircraft event causality in this paper, it is evident that *A*_01_ is the most common event type, and *H*_02_ holds significance in ensuring aircraft safety.These nodes include *A*_02_, *A*_03_, *M*_18_, *M*_21_, *H*_30_, *M*_21_, *H*_45_, and *M*_01_, exhibit higher degrees than other nodes, indicating more frequent association rules with other nodes. This emphasizes the need for specific attention and strict control of these nodes in civil aviation safety management. Additinally, the results show that improper energy allocation, poor conflict resolution ability, inadequate onsite management duties, adoption of a luck mentality, and occurrence of controller oversight are highly correlated with general unsafe events, and failure to rectify incorrect recitation is notably correlated with general incidents, while inadequate manual promotion, lack of conflict judgement and insufficient safety management are strongly correlated with serious incidents. Therefore, in actual aircraft operations, it is essential to prioritize the aforementioned nodes. Prompt action should be taken upon detecting tendencies towards the occurrence of these nodes to prevent the occurrence of unsafe incidents.

Given the importance of ensuring aviation operational safety and preventing events, learning from past events is crucial. However, due to limitations in data sampling and the finite cognitive understanding of manually labelled experiences, it is possible that the results of machine learning might possess directional bias. Therefore, during the next phase of research, efforts will focus on expanding the dataset and employing human experiences more systematically, possibly along the direction of deep learning methodologies.

## Data Availability

Data sets generated during the current study are available from the corresponding author on reasonable request, but restrictions apply to the availability of these data, which were used under license for the current study, and so are not publicly available.

## Abbreviations

NaN: Not a number
TF–IDF: Term frequency–inverse document frequency
HFACS: Human factor analysis and classification system
